# Proof of concept of an accelerometer as a trigger for unilateral diaphragmatic pacing: a porcine model

**DOI:** 10.1186/s12938-023-01119-6

**Published:** 2023-05-31

**Authors:** Tobias Kratz, Roman Ruff, Timo Koch, Anne-Sophie Kronberg, Johannes Breuer, Boulos Asfour, Ulrike Herberg, Benjamin Bierbach

**Affiliations:** 1 Department of Pediatric Cardiology, University Hospital Bonn, Venusberg-Campus 1, 53127 Bonn, Germany; 2 Fraunhofer IBMT, Institute for Biomedical Engineering, Sulzbach, Germany; 3 Department of Pediatric Cardiac Surgery, University Hospital Bonn, Bonn, Germany; 4 Department of Pediatric Cardiology, University Hospital Aachen, Aachen, Germany

**Keywords:** Accelerometer, Diaphragm excursion, Unilateral diaphragm paralysis, Unilateral diaphragm pacemaker, Fontan

## Abstract

**Background:**

Unilateral diaphragmatic paralysis in patients with univentricular heart is a known complication after pediatric cardiac surgery. Because diaphragmatic excursion has a significant influence on perfusion of the pulmonary arteries and hemodynamics in these patients, unilateral loss of function leads to multiple complications. The current treatment of choice, diaphragmatic plication, does not lead to a full return of function. A unilateral diaphragmatic pacemaker has shown potential as a new treatment option. In this study, we investigated an accelerometer as a trigger for a unilateral diaphragm pacemaker (closed-loop system).

**Methods:**

Seven pigs (mean weight 20.7 ± 2.25 kg) each were implanted with a customized accelerometer on the right diaphragmatic dome. Accelerometer recordings (mV) of the diaphragmatic excursion of the right diaphragm were compared with findings using established methods (fluoroscopy [mm]; ultrasound, M-mode [cm]). For detection of the amplitude of diaphragmatic excursions, the diaphragm was stimulated with increasing amperage by a cuff electrode implanted around the right phrenic nerve.

**Results:**

Results with the different techniques for measuring diaphragmatic excursions showed correlations between accelerometer and fluoroscopy values (correlation coefficient 0.800, *P* < 0.001), accelerometer and ultrasound values (0.883, *P* < 0.001), and fluoroscopy and ultrasound values (0.816, *P* < 0.001).

**Conclusion:**

The accelerometer is a valid method for detecting diaphragmatic excursion and can be used as a trigger for a unilateral diaphragmatic pacemaker.

## Background

The diaphragm has an important role in spontaneous breathing. In particular, inspiration and resting breathing depend on diaphragmatic excursion and contraction [1, 2], a dependence that is especially relevant for small children [3]. For this reason, loss of diaphragmatic function may lead to various forms of dysfunctional breathing, including the need for mechanical ventilation [2]. Diaphragmatic function can be disturbed at different levels. The central nervous system and the phrenic nerve may be damaged or affected (e.g., by surgical injury or trauma), or damage at the neuromuscular junction or the muscle directly may lead to impaired respiration [4]. In children with congenital heart disease, especially univentricular hearts, unilateral diaphragmatic paralysis after heart surgery is prominent in postoperative respiratory disturbance. These patients undergo a multi-step palliation [5], in which the upper and lower venae cavae are connected to the pulmonary arteries so that the oxygen-depleted blood flows passively to the lungs. Spontaneous respiration is important in the pulmonary arterial flow and for the whole Fontan circulation [6–9], and unilateral diaphragmatic paralysis leads to impairment of both [10, 11].

To date, the only diaphragmatic pacing systems used clinically stimulate both sides of the diaphragm and require an external stimulator. These systems are used in the treatment of spinal cord injury, amyotrophic lateral sclerosis, and Undine syndrome. A diaphragm pacemaker exclusively for unilateral diaphragmatic paralysis does not exist. Attempts have been made to use bilateral systems for these patients [12], but only Kaneko et al., in an experimental trial, have achieved unilateral diaphragm stimulation based on thorax impedance of the contralateral side [13]. The sole available technically feasible approach is stimulation with a fixed rate without sensing or triggering [14]. The first positive results have been achieved for bilateral diaphragm pacemakers in the context of animal experiments [15].

In view of the limitations of current options, it seems obvious that a technical solution is needed that allows triggering of the diaphragmatic excursion and thus unilateral diaphragmatic pacing. The aim of our research is to develop a unilateral diaphragmatic pacemaker for pediatric cardiac patients with unilateral diaphragmatic paralysis to restore a complete return of diaphragm function. Stimulation of the paralytic side should be triggered based on the non-paretic side. In the current study, we investigated whether an accelerometer is a suitable tool for this purpose.

Fluoroscopy [16] and ultrasound (M-mode) [17] are two established methods for evaluating diaphragm excursion, and we compared the results of accelerometer measures against these two approaches and analyzed correlations among them.

## Results

### Diaphragm excursion

**Fig. 1:**
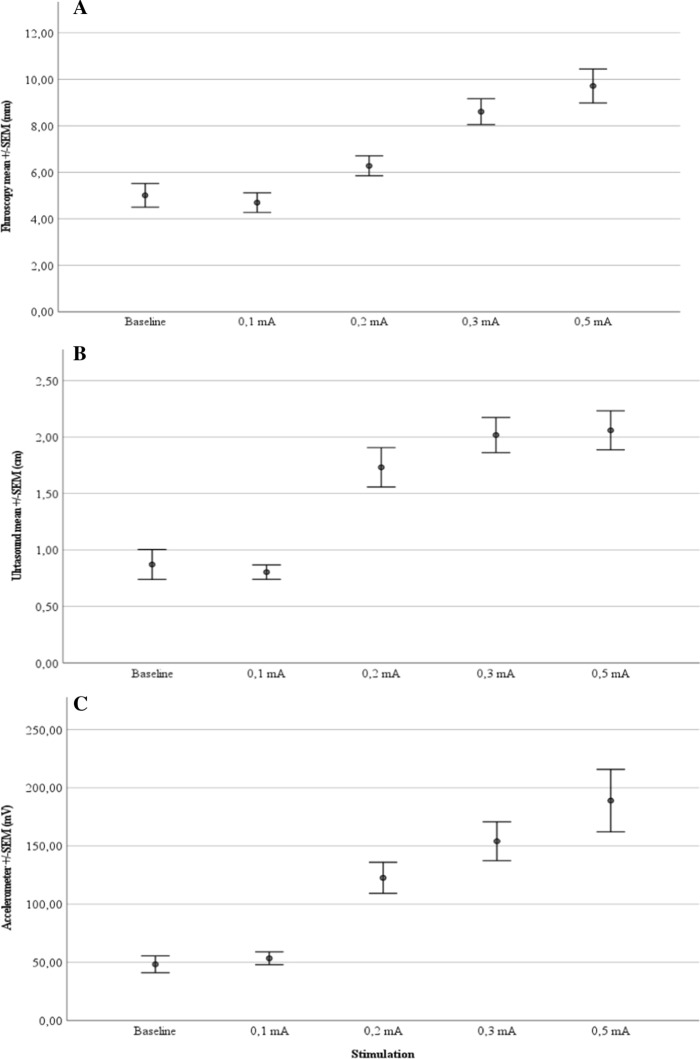
Diaphragmatic excursion (all mean ± standard error of the mean; SEM) as measured by **A** fluoroscopy, **B** ultrasound, and **C** accelerometer under stimulation of the phrenic nerve at different amplitudes (*n* = 7)

Baseline measurements demonstrated right hemidiaphragm excursion of 5.01 ± 0.51 mm (*n* = 7) by fluoroscopy, 0.87 ± 0.13 cm (*n* = 7) by ultrasound, and 48.33 ± 7.23 mV (*n* = 7) by accelerometer. The first significant increase in diaphragmatic movement on the right side could be measured by ultrasound (1.73 ± 0.17 cm, *P* = 0.011) and accelerometer (122.62 ± 13.36 mV, *P* = 0.007) at stimulation with 0.2 mA. The first significant difference by fluoroscopy (8.61 ± 0.55 mm, *P* = 0.003) could be detected at 0.3 mA. All measurements showed a continuous increase of the mean value between stimulation with 0.1 mA to the maximum stimulation with 0.5 mA. All three measurement methods offered reliable detection of any diaphragmatic excursions (Fig. 1).

### Correlations

**Fig. 2:**
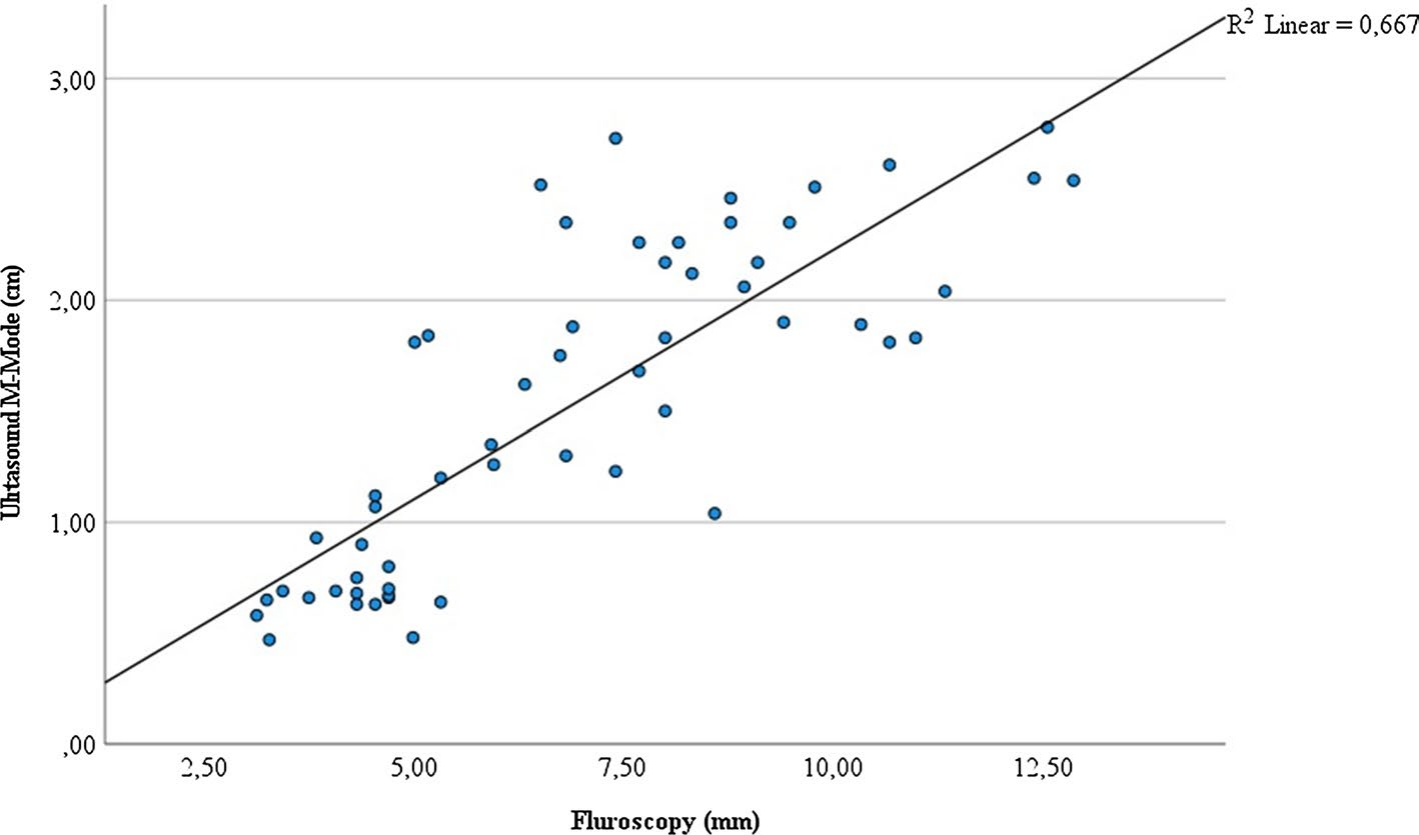
Correlation of measures of right diaphragmatic movement by fluoroscopy (mm) and ultrasound M-mode (cm) (*n* = 7)

**Fig. 3:**
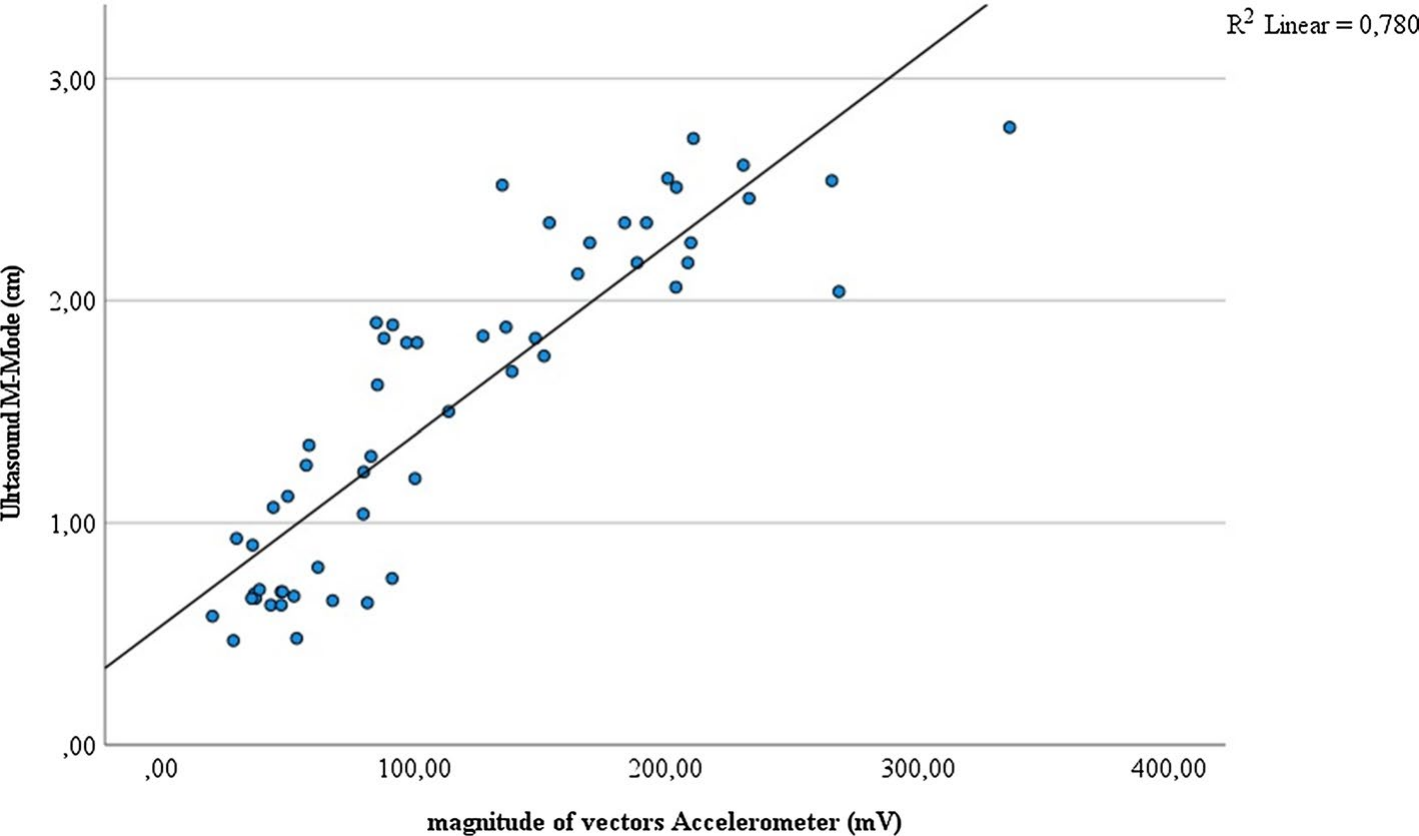
Correlation of measures of right diaphragmatic movement by accelerometer (mV) and ultrasound M-mode (cm) (*n* = 7)

**Fig. 4:**
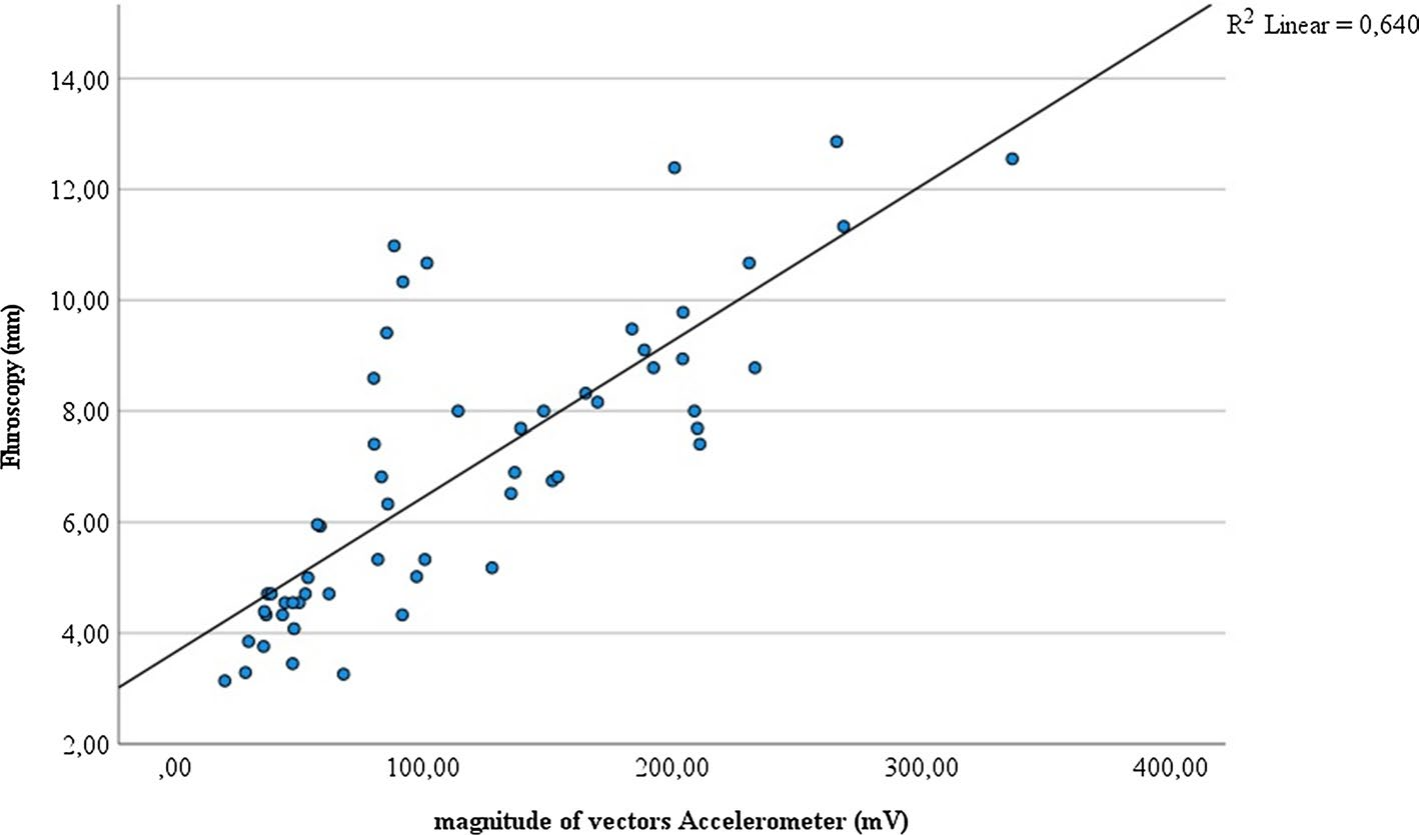
Correlation of measures of right diaphragmatic movement by fluoroscopy (mm) and accelerometer (mV) (*n* = 7)

All correlation analyses of measurements obtained with the three techniques yielded significant correlations, with a correlation coefficient of 0.816 (*P* < 0.001) between fluoroscopy and ultrasound measurements (Fig. 2), 0.883 (*P* < 0.001) between accelerometer and ultrasound measurements (Fig. 3), and 0.80 (*P* < 0.001) between fluoroscopy and accelerometer values (Fig. 4). These results suggest strong linear correlations of these measurements.

## Discussion

Our results demonstrate that diaphragm movement can be reliably recorded with implanted accelerometers. As with clinically established imaging techniques, this system is suitable for determining the excursion of the diaphragm, with accelerometer-based measurement showing a linear correlation with fluoroscopy (*P* < 0.001) and ultrasound (*P* < 0.001) Measurements.

### Unilateral diaphragmatic pacemaker in Fontan patients

Unilateral diaphragmatic paralysis leads to deterioration of the Fontan circulation [11], as well as to reduced perfusion of the pulmonary artery on the paretic side [10]. Currently, no technique allows unilateral diaphragmatic pacing. The accelerometer accurately detects the movement of the diaphragm, so this signal might act as a trigger for unilateral diaphragm pacing. For these reasons, a contralateral diaphragmatic pacemaker triggered by an accelerometer appears to be a potential therapeutic option for Fontan patients with unilateral diaphragmatic palsy.

### Use of the accelerometer in bilateral diaphragmatic pacemakers

Accelerometer-based sensor electronics could be implanted at the highest point of each hemidiaphragm during the implantation of a bilateral thoracic diaphragmatic pacemaker in patients with hypoventilation syndrome (Undine syndrome) [18], to detect the autonomous diaphragm movement. So, an accelerometer may serve as an upgrade to the currently available systems for bilateral diaphragmatic pacing. At the moment, in Undine syndrome patients the bilateral diaphragm pacing most reliably is conducted by direct phrenic nerve stimulation. An accelerometer could be used to check the functionality of the diaphragmatic pacemaker as part of tests/monitoring, with no radiation exposure, in contrast to fluoroscopy. In addition, an implantable accelerometer could be used as a trigger in patients with hypoventilation syndrome to establish a closed-loop system. Hypoventilation or apnea can occur during waking hours as well as during sleep [18, 19], and these apneas with the associated lack of diaphragm movement could be detected by the accelerometer and lead to stimulation.

### Use of the accelerometer in animal experiments

An implantable acceleration sensor also could be used in animal experiments involving the question of diaphragm excursion, especially those that investigate diaphragm excursion over a longer period of time in connection with diseases of the diaphragm. All existing methods for evaluating dynamic diaphragm movement—fluoroscopy [16], ultrasound diagnostics using M-mode [17], and dynamic magnetic resonance imaging [20]—show limitations. With ultrasound, limitations include a restricted field of view, the possible impairment of lung air or bowel gas superimposition, and close reliance on operator expertise. Especially in the left half of the diaphragm, ultrasound is limited by the position of the spleen [21, 22]. Fluoroscopy, which is usually used as a gold standard, entails a high radiation exposure [23]. MRI imaging offers the possibility of a three-dimensional representation but is time-consuming and cost-intensive [22].

## Limitations

The limitations of this study include the lack of representation of physiological respiratory parameters in relation to diaphragmatic excursions. Because the experiment was performed with the thorax open, these data could not be validly measured. The study also was relatively brief, so that no conclusions can be drawn about the long-term course of measuring diaphragmatic movement with an accelerometer. These questions should be addressed in additional studies. Furthermore, in our test, the accelerometer was still powered externally, and data acquisition was external. For future applications, these features would need further development into a fully implantable and battery-operated system.

## Conclusions

The accelerometer is a valid method for detecting diaphragmatic excursions as a possible trigger for a unilateral diaphragmatic pacemaker.

## Methods

Experiments were conducted in accordance with the German Animal Welfare Act and its subsequent statutory acts, which are in accordance with the Council of Europe Convention ETS 123. The competent state agency, the State Office for Nature, Environment and Consumer Protection North Rhine-Westphalia, approved the study (permit: 81-02.04.2020.A392). Our study complied with the Animal Research: Reporting of In Vivo Experiments guidelines 2.0.

### Animal preparation and instrumentation

Seven pigs were examined and had a mean weight of 20.7 ± 2.25 kg. Pigs were supplied in-house by the Agriculture Faculty, Rheinische Friedrich-Wilhelms-University Bonn, Königswinter-Vinxel, Germany, were of conventional microbiologic status, and had an acclimatization period of 3 days at our facility.

Premedication before the operation consisted of intramuscularly administered ketamine (20 mg/kg; WDT, Garbsen, Germany) in combination with azaperone (2 mg/kg; Richter Pharma, Wels, Austria) and atropine (0.02 mg/kg; B. Braun, Melsungen, Germany). After adequate sedation was achieved, venous access was implemented with a 1.1-mm outer diameter Jelco® catheter (Smith Medical, Grasbrunn, Germany) in one of the ear veins. Anesthesia induction consisted of piritramide (0.5 mg/kg; Hameln Pharma, Hameln, Germany) and propofol (10 mg/kg; CP Pharma, Burgdorf, Germany). The administration of muscle relaxants was explicitly dispensed with in this experiment. We secured the airway via endotracheal intubation using a straight size 4 Miller blade with a 5.0-mm internal diameter curved, micro-cuffed endotracheal tube (Avanos, Hamburg, Germany). For invasive ventilation, we used a Servo-i (Maquet, Rastatt, Germany) with synchronized intermittent mandatory ventilation with a frequency of ~ 15/min, an inspiratory pressure of 15 cmH_2_O, and a positive end-expiratory pressure of 5 cmH_2_O. Anesthesia was maintained with continuous intravenous infusion of propofol (1–5 mg/kg) via a Perfusor®Space (B. Braun, Melsungen, Germany) and continuous intravenous infusion of piritramide (0.2–0.5 mg/kg), supplemented by occasional single doses of ketamine (5–10 mg/kg) or midazolam (0.5 mg/kg; B. Braun, Melsungen, Germany). We monitored the depth of the total intravenous anesthesia with the Narcotrend system via a CompactM-monitor for intraoperative use (Narcotrend, Hannover, Germany) using needle electrodes (Neuroline Twisted Pair Subdermal, 12 × 0.4 mm, Ambu, Ballerup, Denmark) placed at the standard positions according to the manufacturer’s instructions. During the procedure, swine had the electroencephalographic Kugler-stadium [24] D0. This stage corresponds to general anesthesia.

We monitored the animals using an Infinity C500 monitor (Dräger, Lübeck, Germany) for electrocardiogram, pulse oximetry, and invasive blood pressure. For capnometry, we used a Datex-Ohmeda S/5 (Datex-Ohmeda, Duisburg, Germany). We placed an arterial line (2.7 French leadercath, Vygon, Aachen, Germany) in the right femoral artery for continuous blood pressure monitoring and a three-lumen central line (5.5 French, Teleflex, Fellbach, Germany) in the right femoral vein. Regular blood samples were taken to check pH, pCO_2_, pO_2_, electrolytes, and hemoglobin. In addition, we placed a 10 Charrière transurethral catheter (Asid Bonz, Herrenberg, Germany) to monitor urine output. Temperature was measured with a 9 French rectal probe (Smiths Medical, Grasbrunn, Germany). Maintenance fluids were infused at a rate of 40–60 mL/kg/h using Ionosteril 1/1 (Fresenius Kabi, Bad Homburg, Germany).

At the end of the experiment, animals were euthanized using T61® (tetracaine/mebezonium/embutramide; Intervet, München, Germany) at a dose of 0.5 mL/kg.

### Technical set-up

#### Accelerometer

**Fig. 5:**
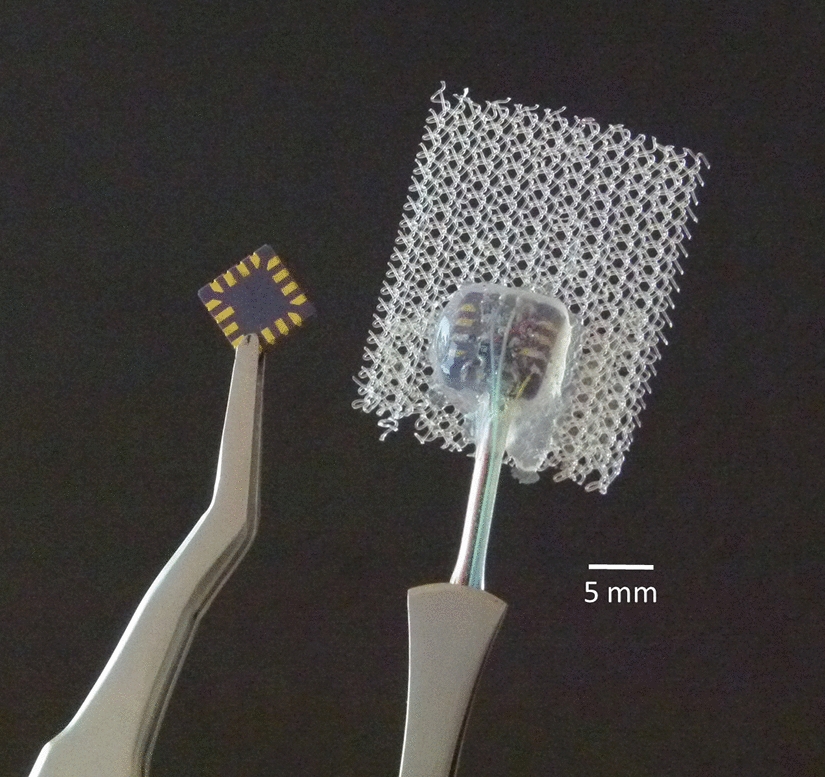
Bare accelerometer IC (left) and assembled and encapsulated sensor with surgical mesh for fixation (right)

We used an MXR9500 3D accelerometer ICs (MEMSIC Inc., Andover, MA, USA) to record the movement of the diaphragm on the right side. The accelerometer converts diaphragmatic motion proportionally into a change of voltage. These sensors are particularly suitable because of their resolution of 1 mg_0_ (*g*_0_ = 9.81 m/s^2^, standard acceleration due to gravity), high sensitivity (500 mV/g_0_), small dimensions (7 × 7 × 1.8 mm^3^), and low requirement for external circuitry. The additional electronic components were soldered directly onto the IC to reduce the volume of the assembly. A silicone tube with an outer diameter of 1.96 mm (Silastic Rx-Medical Grade tubing, Dow Inc., Midland, MI, USA) bundled the leads, and the entire sensor was mechanically fixed with epoxy resin and finally encapsulated with silicone (Silibone MED ADH 4300 RTV, Bluestar Silicones, Ventura, CA, USA). A polypropylene surgical mesh (Angimesh 9, Angiologica B.M. S.r.l., Pavia, Italy) was glued to the sensor, allowing the surgeon to fix the sensor to the implantation site (Fig. 5). An external power supply provided 3 V to the sensors. The three analog outputs of each sensor were connected to six analog single-ended inputs of the bioamplifier (Powerlab 16/35, ADInstruments Ltd, Sydney, Australia).

All inputs had a sensitivity of 156 µV at ± 5 V full range. Depending on the initial sensor position relative to gravitational acceleration, all sensors were adjusted to an individual offset to achieve maximum signal representability. All channels were low-pass filtered at 50 Hz to suppress electrical hum.

#### Cuff electrode

**Fig. 6:**
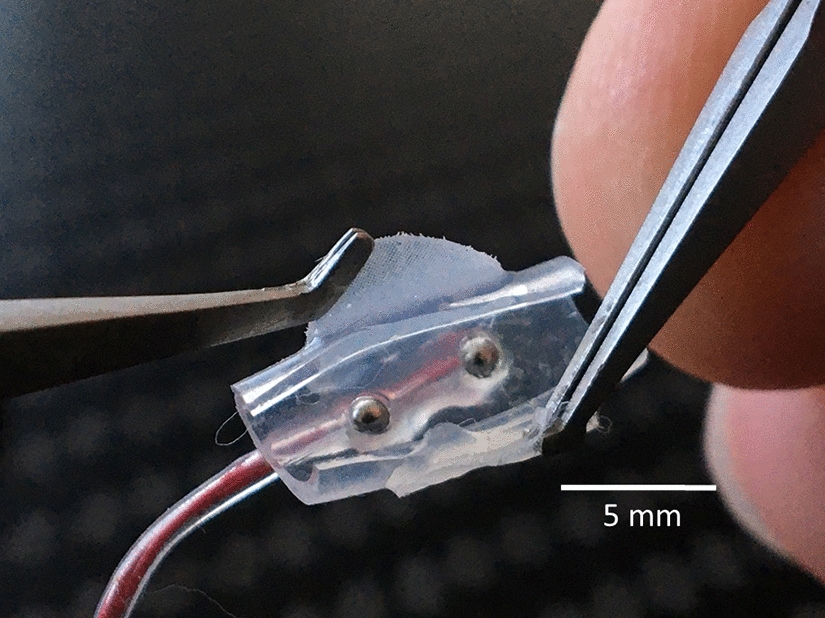
Cuff electrode

We developed and manufactured a simple cuff electrode design for short-term acute experimental setups. Two medical-grade stainless steel electrode contacts were 1.2 mm in diameter and bonded into a silicone tube (Sil-Tec®, 602-305, Technical Products Inc. of Georgia, Lawrenceville, GA, USA) with a center-to-center spacing of 5 mm. This tube has an inner diameter of 1.98 mm, matching the contacted nerve, and is incised over the entire length of 11 mm. Two glued-on wings made of silicone foil with a thickness of 175 µm (NA 501-1, NAGOR, Glasgow, Scotland, UK) allowed for easy handling and positioning on the nerve (Fig. 6).

### Intervention set-up

**Fig. 7:**
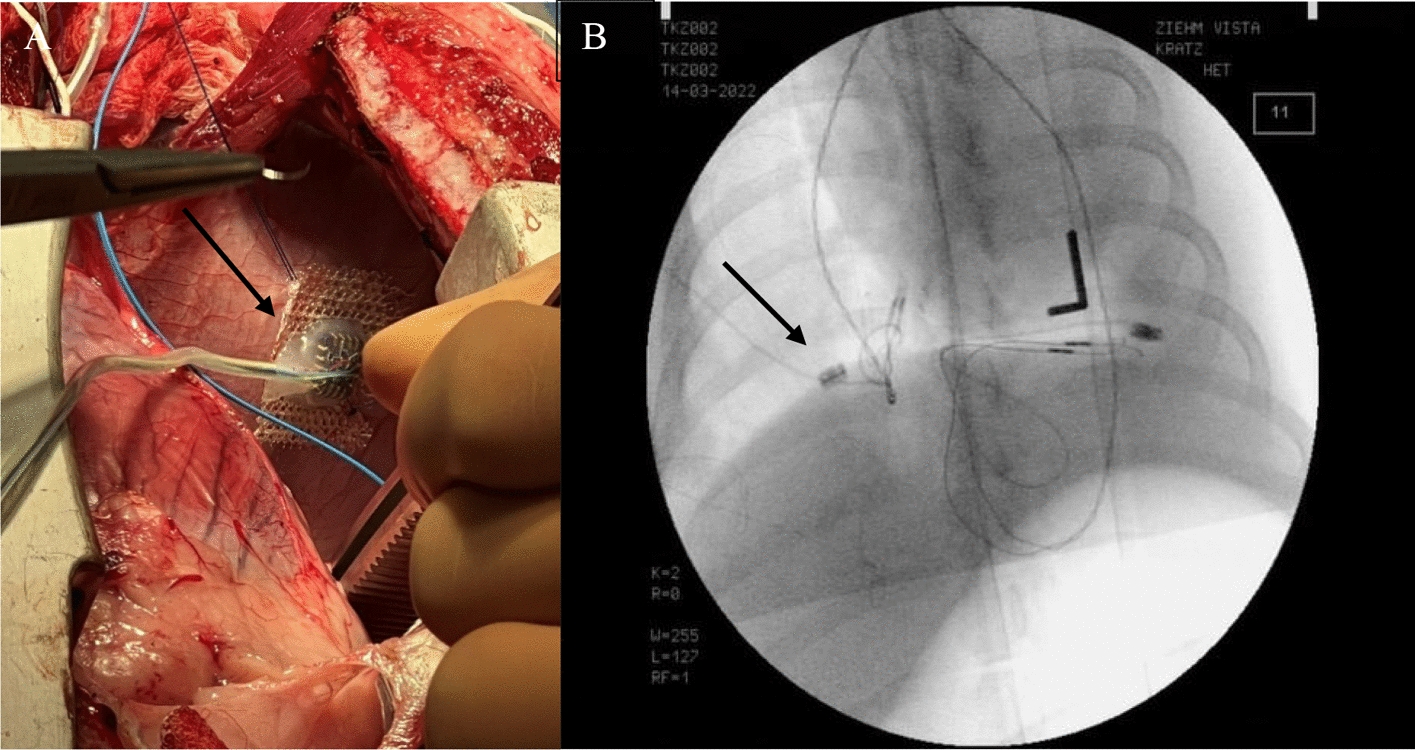
**A** Implantation of the accelerometer (→) on the right diaphragmatic dome. **B** Fluoroscopy to assess the position of the accelerometers (→)

After the pigs were placed in the supine position, a median sternotomy was performed. The chest remained open during the entire experiment. The pleura was opened, and the diaphragm and the right phrenic nerve carefully circumferentially exposed. Subsequently, the silicone-cuff electrode (Fig. 6) was placed at the intended position of the stimulator electrode at the level of the inferior vena cava. To ensure adequate electrode position, a limited neurolysis of around 20 mm was performed by sharp dissection taking care to injure neither the phrenic nerve nor its nutritive vessels. After final positioning of the stimulator electrode its lines were secured to the skin with simple stitches of a size 0 polyester fiber suture. The accelerometer was then implanted at the highest point of the right diaphragmatic dome (Fig. 7).

The position was checked by fluoroscopy (Ziehm, Nürnberg, Germany). The cuff electrode was implanted at the right phrenic nerve. For current-controlled direct nerve stimulation by means of a cuff electrode, we used a FE180 stimulator (ADInstruments Ltd, Sydney, Australia). To provoke different patterns of diaphragmatic movement, we varied the amplitude (i.e., 0.1, 0.2, 0.3, 0.5 mA) and kept the pulse width (200 µs), frequency (30 Hz), and stimulation duration (300 ms) fixed.

### Measurement method

**Fig. 8:**
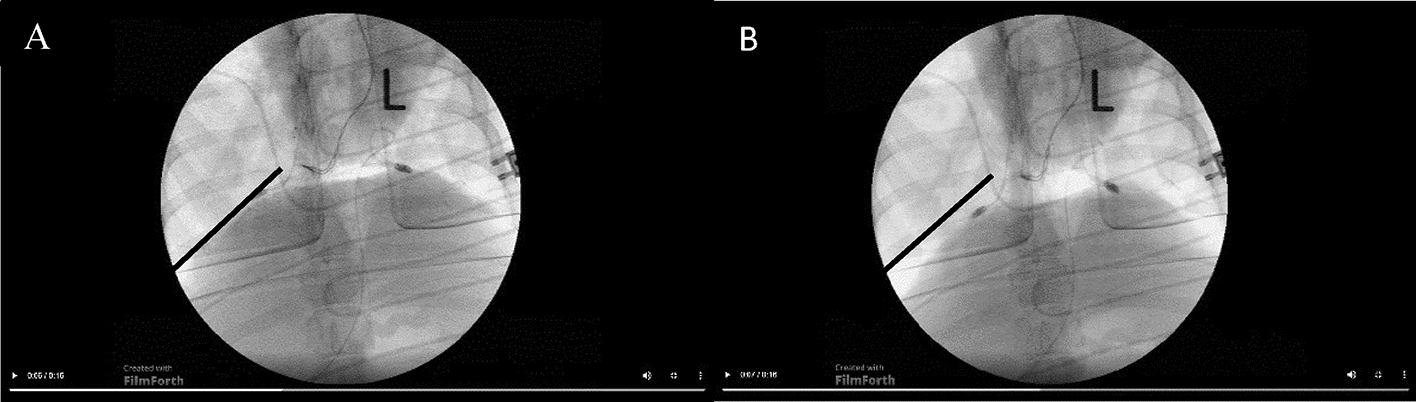
**A** diaphragm in expiration and **B** in inspiration. The line represents an example of the measurement

To evaluate the diaphragm excursion by fluoroscopy (Fig. 8), a virtual tangent was drawn across the highest point of the diaphragm, and the diaphragm excursion was measured at a right angle in millimeters. At minimum of three diaphragmatic excursions were recorded, and the mean value was taken.

**Fig. 9:**
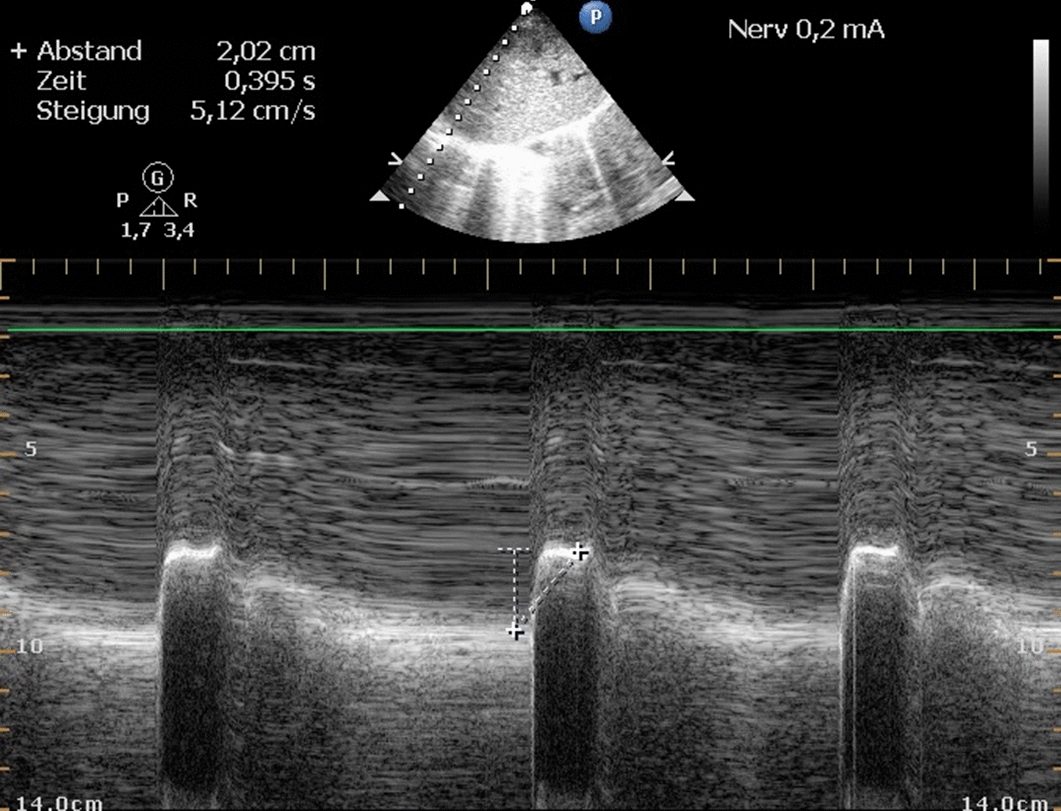
Measurement of diaphragm movement by ultrasound (M-mode) under stimulation of the phrenic nerve with 0.2 mA (three measurements were made for data recording, from which the mean value was calculated)

Diaphragmatic excursion by ultrasound was assessed with a subxiphoid plane and in M-mode as described by Epelman et al. [25]. For this purpose, we used an ultrasound Philips HD 15 (Philips, Amsterdam, The Netherlands) with an S5–2.5 MHz sector array transducer (Fig. 9).

**Fig. 10:**
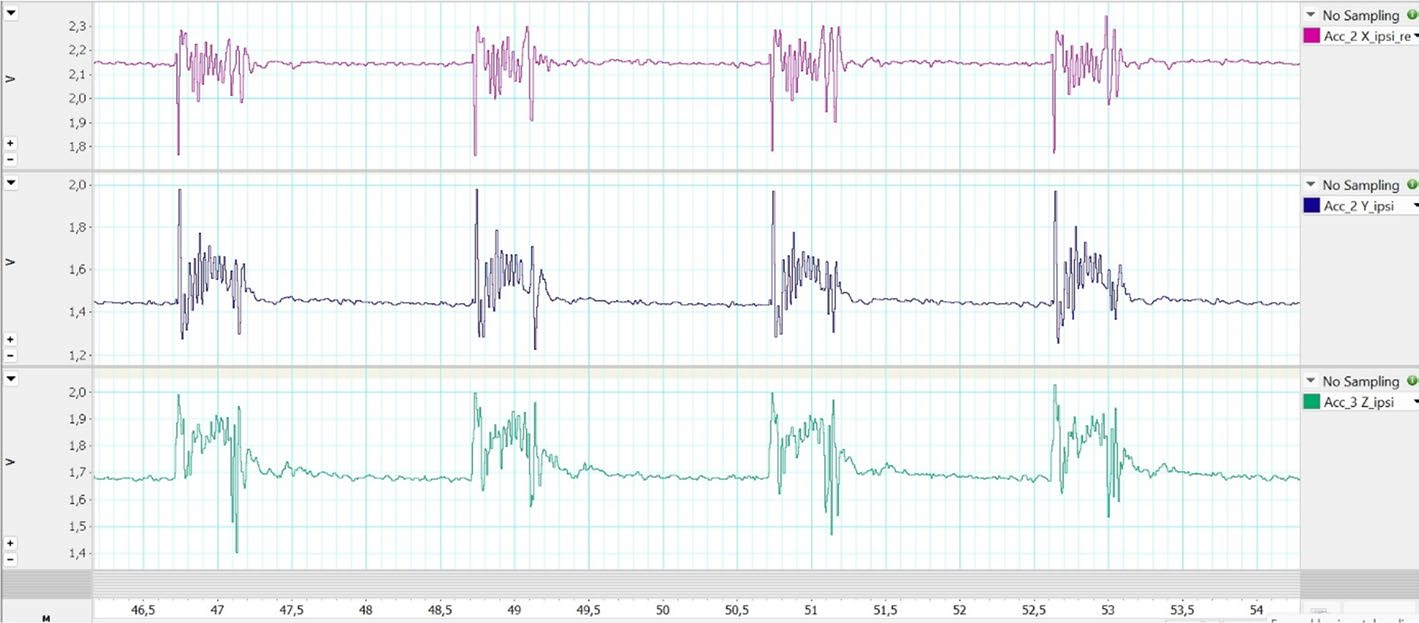
Illustration of the individual vectors (*x*, *y*, and *z* axes) of the accelerometer. Three measurements were always averaged for statistical evaluation

The diaphragm excursion was displayed in three planes by using the accelerometer (Fig. 10). For the final representation of the diaphragm excursion by means of the accelerometer, the individual accelerometer vectors (*x*, *y*, *z*) were taken, and the magnitude of vectors was calculated to represent a movement in the three-dimensional space. All measurements were taken simultaneously.

Parallel evaluation of the left diaphragm was not performed because of the more difficult imaging window involved and the difficult surgical access to the left phrenic nerve. Baseline measurements were taken under the ventilation setting with an inspiratory pressure of 15 cmH_2_O, a positive end-expiratory pressure of 5 cmH_2_O, and a breathing rate of 15/min.

### Data analysis

Data are presented as mean ± standard error of the mean. Statistical analysis was performed with IBM SPSS Statistics Version 28. Three measurements were always averaged for statistical evaluation. The statistical significance of changes from baseline values within each group was tested with analysis of variance (ANOVA) for repeated measures. Differences between groups were analyzed by one-way ANOVA comparing several groups. When values did not show a normal distribution, ANOVA for nonparametric values (Kruskal–Wallis test) was used. A linear regression calculation was performed to test the correlation of accelerometer, ultrasound M-Mode, and fluoroscopy values. Correlation coefficients (*r*) ≥ 0.7 and ≤ − 0.7 were assumed to indicate a correlation between these variables. Statistical significance was accepted at *P* ≤ 0.05 after multiple testing.

## Data Availability

The datasets used and/or analyzed during the current study are available from the corresponding author on reasonable request.
